# The effect of ageing on phenotype and function of monocyte-derived Langerhans cells

**DOI:** 10.1111/j.1365-2133.2011.10313.x

**Published:** 2011-07

**Authors:** S Ogden, RJ Dearman, I Kimber, CEM Griffiths

**Affiliations:** Dermatology Centre, Salford Royal NHS Foundation Trust, University of Manchester, Manchester Academic Health Science CentreManchester M6 8HD, U.K.; * Faculty of Life Sciences, University of ManchesterManchester M13 9PT, U.K.

## Abstract

**Background:**

With increasing age the immune system shows functional decline. In the skin this is associated with an increased incidence of epidermal malignancies and infections. Epidermal Langerhans cells (LCs) act as sentinels of the immune system, recognizing, processing and presenting antigen and inducing T-cell responses. Previous investigations have demonstrated a reduction in the number of epidermal LCs in elderly subjects. Moreover, the ability of LCs to migrate in response to tumour necrosis factor (TNF)-α, but not interleukin (IL)-1β, is significantly impaired in the elderly.

**Objectives:**

To characterize further the changes in LC function that are associated with increasing chronological age, we have evaluated age-related changes in the response of monocyte-derived LCs (mLCs) to IL-1β and TNF-α.

**Methods:**

The phenotype and function of mLCs were compared in six young (≤ 30 years) and six aged (≥ 70 years) healthy individuals using a combination of flow cytometry, cytokine and chemokine array, and a Transwell migration assay.

**Results:**

Monocytes from aged individuals were able to differentiate into LCs. There were no significant differences in expression of activation markers, or in baseline or inducible cytokine secretion, by mLCs derived from aged or young subjects. Furthermore, migration in response to a chemokine ligand, CCL19, was equivalent in both age groups.

**Conclusions:**

These data demonstrate that changes in LC function in the elderly are not associated with changes in systemic dendritic cell phenotype and function. Conditioning of LCs *in situ* by the epidermal microenvironment is likely to be more important.

Ageing of the skin immune system is associated with an increased incidence of epidermal malignancies and infections, a decreased incidence of contact allergy, and the development of autoimmunity. Epidermal Langerhans cells (LCs) act as sentinels of the adaptive immune system, and are adept at processing antigen. Following an external challenge a proportion of epidermal LCs is mobilized and migrates to local skin-draining lymph nodes. While in transit they acquire immunostimulatory properties to facilitate antigen presentation to T cells.^1^ In order to migrate LCs require independent signals from both interleukin (IL)-1β and tumour necrosis factor (TNF)-α. Activated LCs release IL-1β that provides one signal for migration and also triggers production of TNF-α by adjacent keratinocytes. The TNF-α then acts upon LCs in a paracrine fashion to provide a second stimulus for migration.^2^ Previous investigations have demonstrated a reduction in the number of epidermal LCs in elderly subjects.^3^ Moreover, the ability of LCs to migrate in response to TNF-α, but not IL-1β, is impaired in the elderly, the suggestion being that in elderly individuals endogenous IL-1β signalling is perturbed.^4^,^5^

During inflammation and/or injury LCs appear to be replaced by blood-derived precursors, whereas in the steady state they are replaced by local precursors.^6^,^7^*In vitro* CD14+ monocytes cultured in the presence of transforming growth factor (TGF)-β, granulocyte/macrophage colony-stimulating factor (GM-CSF) and IL-4 develop into dendritic cells (DCs) with an LC-like phenotype. These monocyte-derived LCs (mLCs) express CD1a, E-cadherin and langerin,^8^ and stimulation with IL-1β and TNF-α can trigger upregulation of markers of activation.^9^ With the objective of characterizing further the changes in LC function that are associated with increasing chronological age, we have here evaluated age-related changes in the response of mLCs to IL-1β and TNF-α.

## Materials and methods

The study was approved by the local Research Ethics Committee. All participants were required to give written informed consent.

### Culture of Langerhans cells from monocytes

CD14+ monocytes were isolated from peripheral blood mononuclear cells of healthy young (≤ 30 years) and healthy aged (≥ 70 years) volunteers using negative selection with magnetic immunobeads (human monocyte isolation kit; Miltenyi Biotec, Bisley, U.K.). Cells were cultured for 5 days in medium containing IL-4, TGF-β (both at 10 ng mL^−1^) and GM-CSF (at 250 ng mL^−1^) (all R&D Systems, Abingdon, U.K.). On day 6, cells were stimulated with 100 ng mL^−1^ of IL-1β or TNF-α (both R&D Systems) and then cultured for a further 24 h prior to analysis.

### Analysis of monocyte-derived Langerhans cells using flow cytometry

mLCs were stained with HLA-DR (Dako, Stockport, U.K.; clone DK22), CD1a (Dako; clone NA 1/34), CD54 (BD Bioscience, Oxford, U.K.; clone HA58) and CD86 [BD Bioscience; clone 2331 (FUN-1)] or isotype control antibodies followed by fluorescein isothiocyanate-labelled polyclonal goat antimouse immunoglobulin (Dako). Cells were analysed by flow cytometry using FACSCalibur and CellQuest Pro software (Becton-Dickinson, Franklin Lakes, NJ, U.S.A.).

### Cytokine array

Concentrations of IL-1β, TNF-α, IL-10, IL-1Ra, CCL3 and CCL4 were measured in culture supernatants using a Bio-Plex cytokine and chemokine array according to the manufacturer's instructions (Bio-Rad Laboratories, Hercules, CA, U.S.A.) and analysed using a Luminex system (Luminex 100; MiraiBio Hitachi Genetic Systems, South San Francisco, CA, U.S.A.).

### Transwell migration assay

Migration of mLCs was measured using a Transwell assay. Medium containing either vehicle or 200 ng mL^−1^ of CCL19 (R&D Systems) was added to the lower chamber and 2 × 10^5^ of either unstimulated or stimulated (with 100 ng mL^−1^ of IL-1β) mLCs were added to the upper chamber. The cells were incubated for 3 h at 37 °C and fluid was aspirated from the lower chamber and cell counts performed in triplicate using a Casy TT counter (Roche Innovatis, Basel, Switzerland).

## Results

### Comparison of monocyte-derived Langerhans cells from young and aged individuals using flow cytometry

Peripheral blood monocytes were isolated from the blood of six young (age range 20–30 years, mean 25·5) and six old healthy volunteers (age range 70–78 years, mean 74·2). Monocyte yield was equivalent between the age groups.

**Fig 1:**
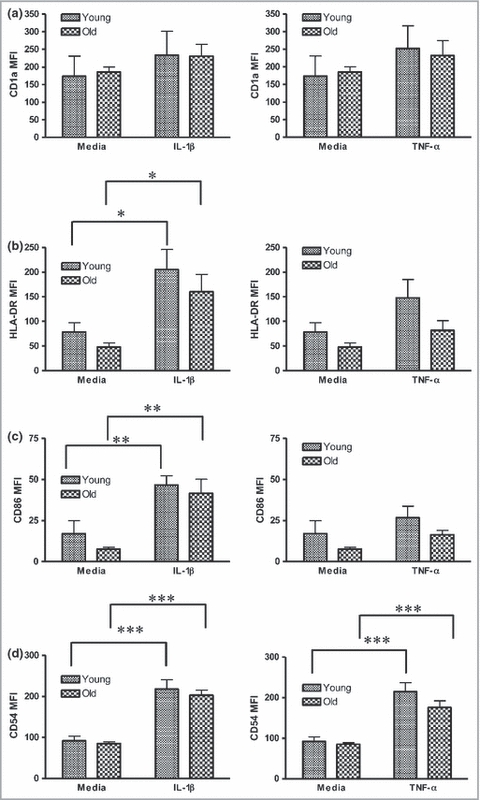
Expression of CD1a, HLA-DR, CD86 and CD54 (a–d, respectively) in monocyte-derived Langerhans cells from young and old subjects (*n* = 6) as measured by mean fluorescence intensity (MFI) of cells cultured with media alone (negative control) and poststimulation with 100 ng mL^−1^ of either interleukin (IL)-1β or tumour necrosis factor (TNF)-α. The effect of age and stimulation upon expression of markers of interest was measured using two-way analysis of variance and Bonferroni post test; data are presented as mean ± SEM. *P <0·05, **P <0·01, ***P <0·001.

The response of mLCs to stimulation with IL-1β and TNF-α was determined firstly by examining the relative levels of expression of markers of LC maturation and activation, recorded as mean fluorescence intensity using analytical flow cytometry. Lipopolysaccharide (LPS) was used as a positive control (data not shown). As expected, a high percentage of both unstimulated and stimulated mLCs expressed CD1a in both age groups Stimulation did not significantly affect the level of expression of CD1a in either age group (Fig. 1a). Most mLCs were HLA-DR+. The level of expression increased significantly in both age groups following stimulation with IL-1β, but not TNF-α (Fig. 1b). Only a relatively small percentage of mLCs was CD86+; however, stimulation with IL-1β led to increased levels of CD86 expression in both groups (Fig. 1c). Most mLCs were CD54+ and stimulation with both cytokines upregulated expression (Fig. 1d). There were no significant differences between the young and old groups in terms of level of expression of markers at baseline or poststimulation.

### Quantification of cytokines and chemokines secreted by monocyte-derived Langerhans cells from young and aged individuals

**Fig 2:**
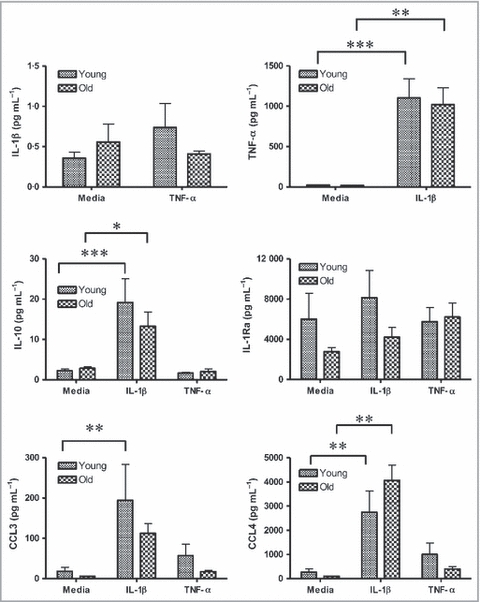
Levels of secreted cytokines and chemokines involved in Langerhans cell (LC) maturation and/or migration were measured in culture supernatants of unstimulated (media) and stimulated [100 ng mL^−1^ interleukin (IL)-1β or tumour necrosis factor (TNF)-α] monocyte-derived LCs derived from young and old individuals (*n =*6) using cytokine bead array. Analysis of the effects of stimulation and age group was performed using two-way analysis of variance and Bonferroni post test; data are presented as mean ± SEM. *P <0·05, **P <0·01, ***P <0·001.

Secretion of cytokines involved in LC maturation and migration was measured in culture supernatants from unstimulated and stimulated mLCs using cytokine bead array (Fig. 2). There were no differences between the young and old groups. The levels of IL-1β were uniformly low and did not increase significantly following stimulation with TNF-α. However, stimulation with IL-1β led to significant increases in the secreted levels of TNF-α, IL-10, CCL3 and CCL4.

### Migration of monocyte-derived Langerhans cells from young and aged individuals in response to a chemokine ligand

**Fig 3:**
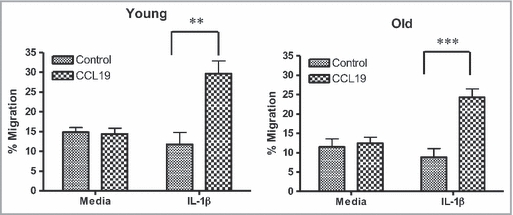
Migratory response of unstimulated (media) and stimulated [100 ng mL^−1^ interleukin (IL)-1β] monocyte-derived Langerhans cells to CCL19 was measured using a Transwell migration assay (*n =*5). The percentage of cells that migrated was calculated and analysis of the effects of IL-1β stimulation and age group was performed using two-way analysis of variance and Bonferroni post test; data are presented as mean ± SEM. **P <0·01, ***P < 0·001.

A Transwell migration assay was used to determine the effect of IL-1β stimulation on the migratory response of mLCs derived from young and old individuals to the chemokine ligand CCL19 (Fig. 3). IL-1β stimulation induced migration in response to CCL19 in mLCs from both age groups. There were no significant differences between the age groups.

## Discussion

The ability of peripheral blood monocytes to differentiate into LCs was not impaired in aged subjects. Consistent with previous reports, a high proportion of the cells expressed CD1a, HLA-DR and CD54, with an upregulation of HLA-DR and CD86 expression observed following stimulation.^8^–^11^ Phenotypically there were no differences in mLCs from young and old individuals. This corresponds to results of earlier studies that have found that the phenotype of monocyte-derived DCs (mDCs) is not affected by the ageing process.^12^,^13^ Although secretion of cytokines by mLCs and mDCs is not directly comparable,^9^ the evidence for age-associated change is variable.^12^–^14^ In this study secretion of chemokines and cytokines involved in LC migration was not impaired; furthermore, there was no increase in secretion of the inhibitory cytokines, IL-1Ra and IL-10, which could have accounted for a reduction in LC migration as seen in aged individuals. Interestingly, the migratory response of LPS-stimulated mDCs from aged individuals to CCL19 was reduced in a study by Agrawal *et al.*,^12^ yet in the current study IL-1β-stimulated mLCs from aged individuals migrated normally in response to CCL19. This may be because of inherent differences in the cell types, as in our study stimulation with LPS did not cause increased migration to CCL19 in either age group (data not shown). Taken together, these data demonstrate that changes in LC function in the elderly are not associated with changes in systemic DC phenotype and function. Thus, conditioning of LCs *in situ* by the epidermal environment is likely to be more important.

**What's already known about this topic?**

- Chronological ageing is associated with impairment of the cutaneous immune response.
- There is an age-associated reduction in Langerhans cell number and function.
- During inflammation/injury Langerhans cells are thought to be replaced by circulating monocyte precursors.

**What does this study add?**

- The phenotype and function of monocyte-derived Langerhans cells are not altered by the ageing process.
- Changes in the epidermal environment are likely to be more important.
